# Pregnancy outcomes following hysteroscopic myomectomy in infertile patients with FIGO type 0–II fibroids: a retrospective study

**DOI:** 10.3389/fmed.2026.1726687

**Published:** 2026-03-25

**Authors:** Nuri Peker, Özge Karaosmanoğlu, Ömür Albayrak, Ayşen Yücetürk, İlke Özer Arslan, Burak Elmas, Bülent Tıraş

**Affiliations:** 1 Acibadem Maslak Hospital, Istanbul, Türkiye; 2 Department of Obstetrics & Gynecology and Reproductive Endocrinology, Acıbadem Mehmet Ali Aydınlar University School of Medicine, Istanbul, Türkiye

**Keywords:** barrier gel, FIGO classification, hysteroscopic myomectomy, *in vitro* fertilization, intrauterine adhesions, myoma

## Abstract

**Background:**

We aimed to evaluate the feasibility and safety of hysteroscopic myomectomy (HM) in infertile patients with International Federation of Gynecology and Obstetrics (FIGO) Type 0, I, and II uterine fibroids.

**Methods:**

This study included infertile patients with FIGO type 0, I, and II uterine fibroids who underwent HM before embryo transfer. After surgery, an intrauterine device (IUD) was placed in the uterine cavity, and an adhesion barrier gel was applied. Two months later, the IUD was removed and a control hysterosalpingography (HSG) was performed. Embryo transfer was carried out in patients without suspected intrauterine adhesions (IUA) on HSG. The primary outcomes were clinical pregnancy and live birth rates, and the secondary outcomes were the complication rate and the incidence of IUA.

**Results:**

Fifty patients were included. Overall, 39 (78%) conceived, and 21 (42%) achieved a live birth. Second-look hysteroscopy was performed in 4 (8%) patients due to suspected IUA. All four patients subsequently conceived; two had a live birth and two had a biochemical pregnancy. Three patients underwent repeat HM because of new myoma formation. After the second HM, HSG showed no IUA in one patient; embryo transfer was performed, but pregnancy was not achieved. In another patient, IUA were detected; hysteroscopic adhesiolysis was performed, and subsequent evaluation showed no IUA, yet pregnancy was not achieved after embryo transfer. In the third patient, a submucosal myoma was detected and a third HM was performed; follow-up HSG showed no IUA, and embryo transfer resulted in a biochemical pregnancy. Overall, 6 (12%) patients developed IUA. No cases of massive bleeding, uterine perforation, or fluid overload were observed.

**Conclusion:**

HM appears to be a safe and reliable method in the treatment of FIGO type 0, I, and II myomas and may improve the pregnancy outcomes when performed before embryo transfer.

## Introduction

Uterine fibroids are benign tumors that originate from the smooth muscle layer of the uterus and are commonly seen in women of reproductive age. They are asymptomatic in most cases and are often diagnosed during routine gynecologic examinations; therefore, the exact prevalence is unknown. However, approximately one in four women at reproductive age experience myomas and they are estimated to occur in more than 70% of the women from menarche to the menopause (1–3). The most common symptom is abnormal uterine bleeding, and fibroids may also cause pelvic pain and infertility. Approximately 5%–10% of patients diagnosed with infertility have uterine fibroids (4). Ultrasonography is the first-line method for diagnosing uterine fibroids because it is cost-effective, widely available, and has high sensitivity and specificity. Defining the size, number, and location of uterine fibroids is essential for determining the appropriate treatment; accordingly, myoma classification is crucial. To date, two classification methods have been proposed: the traditional system and the International Federation of Gynecology and Obstetrics (FIGO) classification system (5). The traditional system is based on the relationship between endometrium and uterine serosa and classifies fibroids as submucosal, intramural, and subserosal (5). The FIGO system includes nine subcategories (types 0–VIII); among these, type 0, I, and II myomas may lead to infertility by affecting endometrial receptivity, gamete transfer, or the hormonal milieu (4, 5). Although the effect of type III myoma on infertility remains controversial, several studies suggest associations with implantation, pregnancy, and live birth rates (6). Treatment options for uterine fibroids include medical and surgical approaches, and hysteroscopic removal is recommended for FIGO types 0, I, and II myomas (6, 7).

Hysteroscopy is a minimally invasive procedure in which a scope is used to inspect the uterine cavity, allowing diagnosis and treatment of intrauterine pathologies within the same session. It is generally considered as safe, reliable, and effective procedure with low complication rates (8–10). Early hysteroscopic complications include excessive bleeding, uterine perforation, and fluid overload (9–12), whereas late-onset complications include intrauterine adhesions (IUA), with reported rates ranging from 31% to 45% (13). However, complication frequency and severity may vary depending on the type of hysteroscopic procedure and fibroid characteristics. The risk of intraoperative bleeding and transurethral resection (TUR)-like syndrome is higher, especially in patients with FIGO type II myomas and/or myomas larger than 3 cm (9–12).

Intrauterine adhesions are serious late-onset complications that may cause amenorrhea, dysmenorrhea, and subfertility. They can be evaluated using hysterosalpingography (HSG), saline infusion sonography (SIS), or hysteroscopy. The reported specificity, sensitivity, and positive predictive value (PPV) of HSG are 80%, 75%–80%, and 50%, respectively. SIS has sensitivity and specificity values similar to those of HSG. Nevertheless, hysteroscopy is considered the reference standard for diagnosing IUA and should be preferred when available; HSG and SIS are alternatives when hysteroscopy is not available (13). Classification of IUA is important, and several systems have been proposed. March et al. divided IUA into three groups based on hysteroscopic findings: mild, moderate, and severe (14). The European Society of Hysteroscopy classified IUA as grades I–IV based on hysteroscopic findings, HSG findings, and clinical symptoms (15). The American Fertility Society reported a similar classification approach based on hysteroscopic and HSG findings (16).

In this retrospective study, we aimed to evaluate pregnancy and live birth rates who underwent hysteroscopic myomectomy (HM) and were administered IUA gel after surgery.

## Materials and methods

This retrospective study was conducted at Acıbadem Maslak Hospital IVF Department between 1 January 2017 and 1 January 2024. Clinical records of 18,000 patients who underwent *in vitro* fertilization (IVF) and 190 patients who underwent hysteroscopic myomectomy (HM) were reviewed. Based on the inclusion criteria, 50 patients were included; all had FIGO type 0, I, or II myomas and no history of myomectomy. Patients with a history of hysteroscopic procedures, including myomectomy, adhesiolysis, or septum resection, were excluded. In addition, factors that may affect IVF outcomes independent of myomas, namely female age > 38 years and male azoospermia, were excluded. The study was conducted in accordance with the Declaration of Helsinki and was approved by the Acıbadem University Institutional Review Board/Ethics Committee (Approval No: 2025/07-56). Written informed consent was waived due to the retrospective nature of the study.

The study procedures were divided into three periods: (1) presurgical, (2) surgical, and (3) postsurgical.

### Presurgical period

Following IVF treatment and embryo cryopreservation, patients were assessed using transvaginal ultrasonography (TVUS). The number, size, and type of myomas were determined according to the FIGO classification system. When TVUS provided insufficient information, SIS was performed to better visualize and determine the myoma location, size, and type. During SIS, a sterile catheter was inserted into the endometrial cavity and 10 mL of sterile saline was injected to distend the uterine cavity and provided a clear, single-layer view of the endometrium. Patients were then scheduled for hysteroscopic surgery on the following day. At our center, the FIGO classification system is routinely used to evaluate the uterine fibroids, and FIGO types 0, I, and II myomas are removed hysteroscopically. No medical pretreatment was administered to reduce myoma size.

### Surgical period

Operative hysteroscopy was performed under general anesthesia in the operating theater. All hysteroscopic procedures in the current study were performed by the same surgeon who has more than 10 years of experience in hysteroscopic surgery. Cervical dilatation was achieved using Hegar dilators, and no cervical ripening medication was administered before surgery. A 22-Fr unipolar resectoscope with a 30° oblique lens (Karl Storz SE, Tuttlingen, Germany) was used to remove fibroids, which were sent for pathological evaluation. In cases where dense IUAs were observed during hysteroscopy, they were excised with a cold knife. After myoma removal, an adhesion barrier gel (MateRegen Gel, Kebomed) was applied into the endometrial cavity, and an IUD (Copper T 380) was inserted as a physical barrier. As a temporary mechanical spacer mainly due to institutional availability and cost considerations, copper was preferred. According to our institutional practice, barrier gel is used as part of a standardized postoperative adhesion-prevention strategy in patients undergoing hysteroscopic surgery. Patients were discharged on the same day. Postoperative treatment included doxycycline 100 mg twice daily for 7 days and Cyclo-Progynova (Bayer Weimar GmbH und Co., KG), 21 days and two cycles.

### Postsurgical period

**FIGURE 1:**
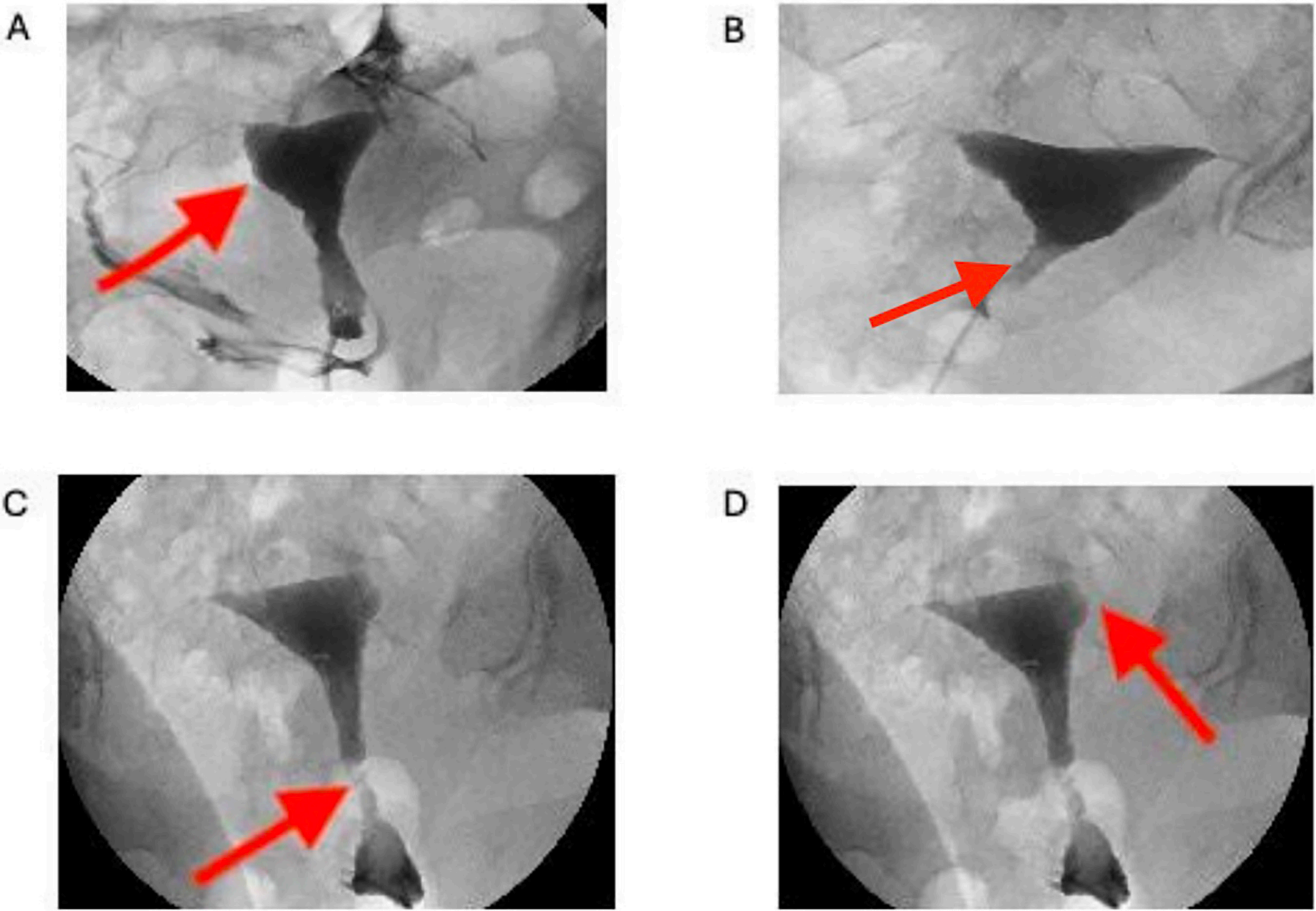
Illustrates the hysterosalpingography (HSG) view of intrauterine adhesions. **(A)** Red arrow demonstrates the intrauterine adhesions at the cornual region of the uterus. **(B,C)** Shows irregular filling defects at the istmic region. **(D)** Represents a filling defect t the left cornual region.

After surgery, the IUD was removed in all patients at the end of the second menstrual period. Within the following days, HSG was performed, and findings were evaluated by the same reproductive endocrinologist with >30 years of experience. Figure 1 illustrates HSG findings consistent with intrauterine adhesions. Subsequent hysteroscopy was performed in patients with suspected IUA. Embryo transfer was planned for patients without suspected IUA.

### Embryo transfer

All patients underwent hormone-replacement frozen embryo transfer (HRT-FET) cycles. A gonadotropin-releasing hormone (GnRH) agonist (Zoladex^®^, 3.6 mg; AstraZeneca, United Kingdom) was administered subcutaneously on day 21 of the preceding cycle for pituitary downregulation. On cycle day 2 or 3, patients underwent a baseline transvaginal ultrasonography. Endometrial preparation was initiated in patients with an endometrial thickness ≤ 5 mm and no evidence of ovarian activity. Oral estradiol valerate (Estrofem^®^ 2 mg; Novo Nordisk, Denmark) was prescribed twice daily for 4 days, three times daily for the subsequent 4 days, and four times daily for an additional 4 days (total: 12 days). This regimen resulted in a total estradiol dose of 8 mg/day before endometrial evaluation. At follow-up, endometrial thickness and pattern were assessed.

### Progesterone support and embryo transfer

Patients with appropriate hormonal levels started progesterone replacement therapy consisting of subcutaneous Prolutex (IBSA Institut, Switzerland) 25 mg twice daily and vaginal Progestan capsule (Koçak Pharma, İstanbul, Turkey) 200 mg three times daily. Embryo transfer was scheduled 6 days after progesterone initiation. On the day of embryo transfer, cycles with serum progesterone < 10 ng/mL were canceled. For serum progesterone levels of 10–20 ng/mL, a single intramuscular (IM) dose of progesterone, Progestan^®^ 50 mg; (Koçak Pharma, Turkey) was administered in addition to routine luteal support. Cycles with serum progesterone ≥ 20 ng/mL proceeded without additional intervention and were included in the study.

In patients under 35 years, a single day-5 embryo was transferred, while in patients over 35 years-old, two day-5 embryos were transferred. According to the Gardner classification, AA, AB, BA, BB, AC, CA, BC, and CB embryos were transferred, while CC embryos were not transferred.

### Statistical analysis

All data were analyzed using SPSS for Windows (version 25; IBM Corp., Armonk, NY, United States). Normality was assessed using the Shapiro–Wilk test. Categorical variables were compared using the chi-square test or Fisher’s exact test, as appropriate. Continuous variables were expressed as mean ± standard deviation (SD), and categorical variables as number (*n*) and percentage (%). Statistical significance was set at *p* < 0.05.

## Results

A total of 50 patients were included. Pregnancy was achieved in 39 (78%) patients following IVF. Among these, 8 (16%) had biochemical pregnancies, 9 (18%) had clinical pregnancies that ended in miscarriage, and 22 (44%) achieved a live birth.

**FIGURE 2:**
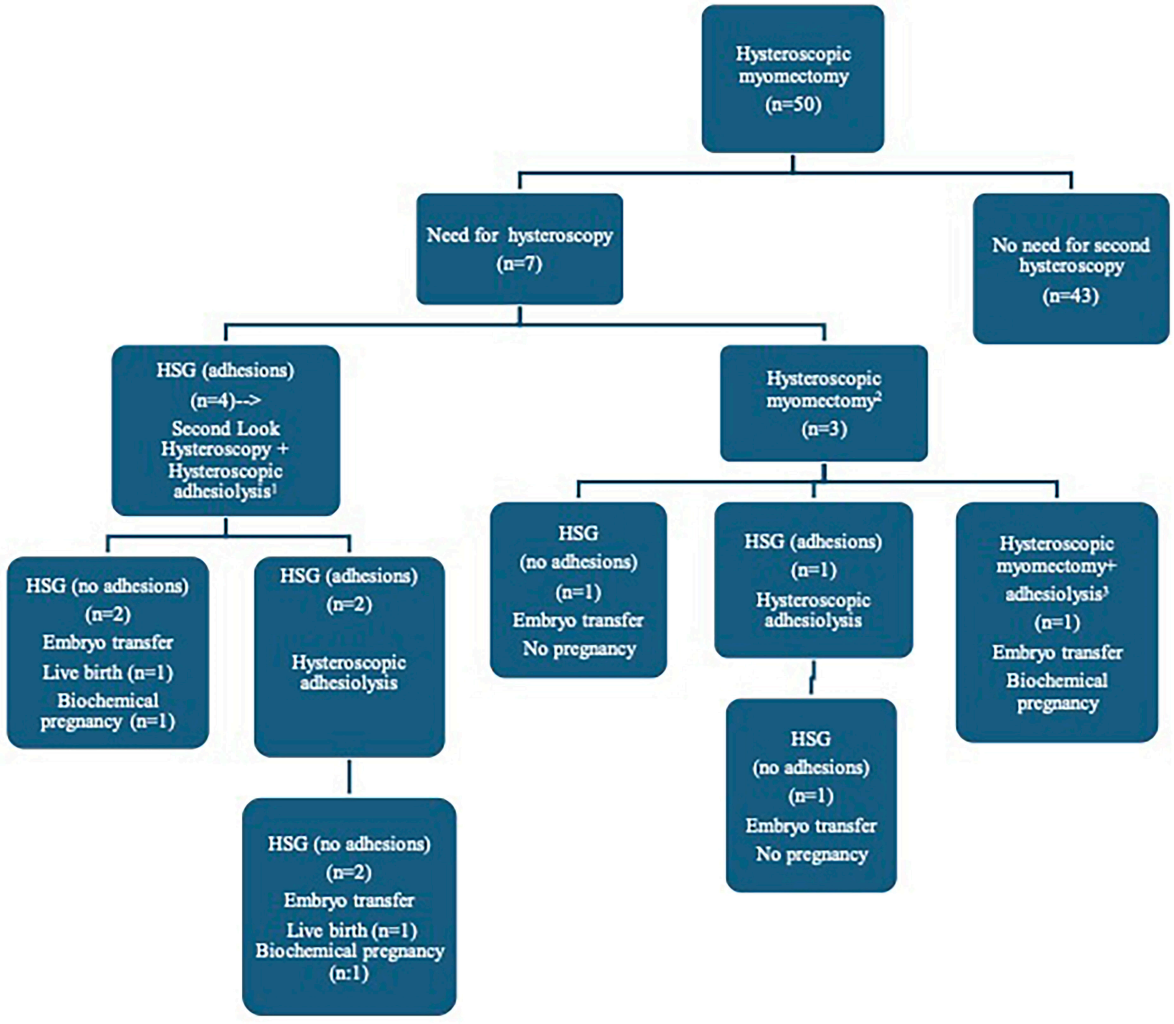
Shows the treatment and follow-up scheme applied to the patients enrolled in the study. The flow diagram gives information about the treatment protocols followed for all patients. (1). A second-look hysteroscopy was performed in four patients after HM due to HSG findings suggesting the presence of intrauterine synechiae. (2). Hysteroscopic myomectomy was performed in three patients due to the presence of newly formed myomas. (3). A third hysteroscopic myomectomy was performed because newly formed myomas were observed. During the procedures, dense intrauterine adhesions were observed, and adhesiolysis was performed. HM, hysteroscopic myomectomy; HSG, hysterosalpingography.

Figure 2 illustrates the diagnostic and treatment protocols followed for all patients. A second-look hysteroscopy was performed in four patients after HM due to HSG findings suggesting the presence of IUA. Embryo transfer was performed after the entire process was completed, and two patients had live birth and the others had biochemical pregnancy.

Three patients underwent a second HM procedure due to new myoma formation. After the repeat procedure, the standard postsurgical protocol was applied, and HSG was performed at the end of the second menstrual period. In one patient, no IUA was detected, and embryo transfer was performed; however, pregnancy was not achieved. In a second patient, IUAs were detected on HSG, and hysteroscopic adhesiolysis was performed; follow-up HSG at 2 months showed no IUA, yet pregnancy was not achieved after embryo transfer. In the third patient, a submucosal myoma was detected, and a third HM was performed. Newly formed myomas were also removed from the cavity during the same session. Two months later, follow-up HSG showed no IUA, and embryo transfer resulted in a biochemical pregnancy.

Overall, 6 (12%) patients developed IUA. No cases of massive bleeding, uterine perforation, or fluid overload were observed.

Table 1 presents patient demographic characteristics. Mean age was 34.18 ± 3.71 years among patients who conceived, and 33.64 ± 3.24 years in those who did not. There were no significant differences in age, body mass index (BMI), and infertility duration.

**TABLE 1:** Demographic characteristics of patients.

Variable	Pregnancy	Number (*n*)	Mean	Std. deviation	Std. error mean
Age	No	11	34.18	3.710	1.119
	Yes	39	33.64	3.240	0.519
BMI	No	11	25.073	5.9621	1.7976
	Yes	39	24.372	4.2014	0.6728
Duration of infertility	No	11	7.27	4.839	1.459
	Yes	39	6.23	4.158	0.666

Table 2 shows the association between myoma number/size and pregnancy rate. No significant association was found between fibroid number or size and the pregnancy outcomes.

**TABLE 2:** The relationship between the number and size of myomas and the pregnancy rate.

Variable	Pregnancy (*n*)			
		Not pregnant *n* (%)	Pregnant, *n* (%)	Total *n* (%)
Number of fibroids	1	7 (19.4%)	29 (80.6%)	36 (72%)
	2	4 (33.3%)	8 (66.7%)	12 (24%)
	3 or more	0 (0%)	2 (100%)	2 (4%)
Size of fibroids (milimeter) (mean)		15.91 ± 3.743	15.08 ± 1.639	–

Table 3 shows pregnancy outcomes by FIGO classification. No significant differences were observed among FIGO types 0, I, and II.

**TABLE 3:** The relationship between the International Federation of Gynecology and Obstetrics (FIGO) classification of the fibroids and the pregnancy rates.

FIGO type		Pregnancy		
		Not pregnant *N* (%)	Pregnant *N* (%)	Total *N* (%)
Type of fibroids (FIGO classification)	Type 0	0 (0%)	4 (100%)	4 (8%)
	Type 1	5 (21.7%)	18 (78.3%)	23 (46%)
	Type 2	4 (21.1%)	15 (78.9%)	19 (38%)
	Type 1 + 2	2 (50%)	2 (50%)	4 (8%)
		11 (22%)	39 (78%)	50 (100%)

## Discussion

In this retrospective cohort of infertile patients undergoing embryo transfer following HM for FIGO type 0–II fibroids, 39 out of 50 (78%) achieved pregnancy, and 21 out of 50 (42%) achieved live birth. IUA were identified in six out of 50 cases (12%), and five out of 50 cases (10%) underwent a second-look hysteroscopy due to follow-up results. There were no major complications during or after the surgery, such as massive bleeding, uterine perforation, or fluid overload. Within the limitations of this study, fibroid size, number, and FIGO type were not significantly associated with IUA incidence or pregnancy outcomes.

Hysteroscopy is a minimally invasive and generally safe procedure that allows evaluation of the intrauterine cavity and treatment within the same session (17, 18). It is considered the gold-standard technique for managing various intrauterine pathologies, including endometrial polyps, fibroids, uterine septum, and IUA (18, 19). The overall complication rate has been reported as 0.95%, with hemorrhage being the most common event, followed by uterine perforation and cervical laceration (9, 12, 18, 19). Distension media overload is a rare yet serious complication (reported incidence < 5%) and may be life-threatening. Late-onset complications include IUA and pelvic infection, which are clinically important because they may impair fertility. IUA may develop secondary to hysteroscopic myomectomy (HM) and may be associated with several factors.

Takasaki et al. evaluated IUA incidence in 217 patients who underwent HM and categorized patients into three groups: (i) a single myoma, (ii) apposing myomas, and (iii) fibroids located far from each other. IUA were more frequent in Group II, suggesting apposing myomas as a potential risk factor for adhesion formation (20). Similarly, Yang et al. reported a higher incidence of IUA after HM in the presence of apposing myomas (21). Bortoletto et al. compared IUA rates after abdominal myomectomy, minimally invasive myomectomy, and HM, and although IUA were more frequent after HM, no significant differences were observed among groups. They also concluded that fibroid number and size were not associated with IUA incidence; however, most patients who developed IUA had myomas extending into the uterine cavity (22). Catena et al. reported that myoma size is a key determinant of complete hysteroscopic removal in a single session (23). As fibroid size increases, the likelihood of repeat surgery may increase, potentially leading to greater endometrial trauma and, consequently, higher IUA risk. Riemma et al. similarly reported a direct relationship between myoma size and operative duration and difficulty, and found that procedure time was significantly longer for type 0 and type I myomas ≥ 3 cm; myoma location and depth also prolonged procedure time (24). Accordingly, fibroid size ≥ 3 cm may indirectly increase IUA risk by increasing the likelihood of repeat surgery and endometrial trauma (24). In the present study, the mean fibroid size was about 15.91 ± 3.743 in not pregnant, while 15.08 ± 1.639 in pregnant group; which is relatively small and may have facilitated complete removal in a single session. This might be explained via the absence of major perioperative complications and the relatively favorable pregnancy outcomes. However, this also restricts generalizability to patients with larger (≥3 cm) fibroids, in whom repeat procedures and endometrial trauma may be more frequent.

Zhang et al. assessed IUA after HM: among 44 patients without prior IUA, 4 (9.1%) had IUA on second-look hysteroscopy. Among nine patients with prior IUA who underwent HM, 5/9 (55.6%) developed IUA; to conclude, they reported that myoma number, size, and the deepest type were not associated with IUA formation; additionally, resection of opposing fibroids in the same session did not increase new IUA formation (23, 25). In a retrospective cohort study, Ikemoto et al. performed second-look hysteroscopy after the second menstrual bleeding and reported no perioperative (massive bleeding, uterine perforation, or water intoxication) or postoperative complications. IUA developed in five patients; those patients had a greater number of myomas removed, longer operative times, and higher intraoperative bleeding volumes. The authors concluded that IUA incidence was significantly associated with the number of enucleated fibroids (24, 26).

In our study, patients were not categorized by fibroid location (apposing vs. non-apposing); however, consistent with the literature, fibroid size, number, and type were not associated with IUA incidence.

Intrauterine adhesions are defined as fibrotic bands within the endometrial cavity, typically resulting from prior surgery or infection (e.g., genital tuberculosis with endometrial involvement). Classification is important because treatment and prognosis are closely related to adhesion severity. Several classification systems have been proposed. March et al. categorized adhesions as mild, moderate, or severe based on the extent of uterine cavity involvement on second-look hysteroscopy (14). Similarly, Hamou et al. performed a second-look hysteroscopy for classifying adhesions and grouped the adhesions as isthmic, marginal, central and severe (27). Valle and Sciarra, Donnez and Nisolle used either hysteroscopy or HSG in classifying adhesions (28, 29). They divided adhesions into six grades based on the location (29). The European Society of Hysteroscopy proposed a four-grade system (grades I–IV) with subtypes incorporating clinical symptoms, HSG findings, and hysteroscopic findings (30). Hysteroscopy is the gold standard for diagnosing IUA, however, 2D (two dimensional) ultrasonography, 3D ultrasonography, hysterosonography, hysterosalpingography should be used in the diagnosis (29). According to the study by Ahmadi et al. it was reported that the IUA mimics filling defects distorting the appearance of the uterine cavity. These filling defects have irregular, multiply angulated shapes and are immobile (31). Amin et al. published an article defining the appearance of intrauterine adhesions (32). Similar with Ahmadi et al. it was reported in the article that filling defects and irregular image of uterine cavity indicates IUA (32).

Numerous preventive strategies, including hyaluronic acid gels (HAG) and intrauterine device (IUD) placement, have been used to reduce IUA after hysteroscopic resection of submucous fibroids. Several studies support the use of cross-linked HAG for IUA prevention. A systematic review and meta-analysis reported that HAG use after HM reduced IUA incidence and increased pregnancy rates (33). Acunza et al. conducted a prospective randomized controlled trial (RCT) evaluating auto–cross-linked HAG after hysteroscopic adhesiolysis and found that adhesion severity and recurrence were significantly lower in patients receiving postoperative HAG (34). Moa et al. conducted an RCT in women with moderate-to-severe IUA (*n* = 306): in the intervention group, HAG was administered twice (immediately after adhesiolysis and again 5–7 days later), whereas no HAG was used in controls; endometrial thickness, implantation rate, and clinical pregnancy rate were significantly higher in the HAG group (35). Huang et al. reported lower IUA recurrence after HM in patients receiving HAG compared with those who did not (12.8% vs. 39.1%, *p* = 0.012), with significantly lower adhesion severity in the HAG group (36). Consistently, Zheng et al. suggested that HAG reduced IUA formation and improved pregnancy rates after intrauterine procedures (37). HAG has also been investigated after procedures such as hysteroscopic polypectomy, septum resection, myomectomy, and adhesiolysis, and after dilation and curettage (D/C) or vacuum aspiration, to prevent IUA. Vatanatara et al. evaluated alginate carboxymethylcellulose HAG after first-trimester vacuum aspiration and reported reduced IUA formation but no significant reduction in adhesion severity (38). Sroussi et al. conducted a multicenter prospective RCT in patients undergoing D/C for miscarriage between 7 and 14 weeks of gestation: HAG reduced IUA rates, but pregnancy rates were not significantly improved (39). Similar findings were reported by Li et al., Can et al. showing significantly lower IUA incidence with HAG after D/C in the first or second trimester (40, 41).

Conversely, some trials have reported no preventive benefit of HAG. Guo et al. conducted a single-center, double-blind RCT evaluating cross-linked HAG in addition to intrauterine balloon insertion and estradiol; second- and third-look hysteroscopies showed comparable IUA incidence between groups, suggesting no reduction in IUA with HAG (33, 42). Zhou et al. conducted a double-blind RCT in 245 patients evaluating IUA recurrence and fertility scores and reported that HAG did not appear effective for preventing IUA (34, 43). Trinh et al. enrolled 200 women diagnosed with IUA after HSG and compared HAG alone, IUD alone, and combined HAG + IUD; adhesion incidence and severity were lower in the HAG group than in the IUD group, and the combined approach showed the best results, although none of the methods significantly improved pregnancy rates (44). Overall, multiple RCTs suggest that HAG after hysteroscopic surgery may improve endometrial thickness/receptivity and reduce IUA incidence and severity.

Intrauterine balloon tamponade is another option used in the prevention of IUA. Yang et al., Shi et al. reported that the incidence of IUA was reduced in patients that underwent intrauterine balloon tamponade after hysteroscopic surgery (45–47). Shi et al. conducted a randomized controlled study with 191 patients who had moderate to severe IUA and underwent hysteroscopic adhesiolysis. The participants were divided into two groups, group (I), the balloon group, whom received intrauterine balloon application at 2^nd^ and 6^th^ week after the surgery and group (II), control group, those did not undergo balloon dilatation therapy. It was reported that overall IUA formation was significantly lower in the balloon group (47). Sun et al. investigated early second-look office hysteroscopy combined with intrauterine balloon dilation in 156 women with IUA. The intervention group underwent balloon dilation 10 days after adhesiolysis and second-look hysteroscopy 20 days after adhesiolysis, whereas controls underwent hysteroscopy 3 months after surgery. The primary outcome was pregnancy rate, which was higher in the intervention group (48.7% vs. 30.1%, *p* < 0.05) (38, 48).

Recent advances in biomedical engineering have increased interest in novel approaches to prevent and treat IUA. In a multicenter study conducted in 2022, Weyers et al. evaluated the safety and effectiveness of an intrauterine degradable polymer film (DPF) for preventing IUA after HM. DPF was inserted into the uterine cavity in 23 patients and fully degraded within 7 days; 20 of 23 patients had no IUA on second-look hysteroscopy (49). In 2024, Fernandez et al. conducted a randomized controlled trial evaluating intrauterine DPF in the management of moderate-to-severe IUA (50). After hysteroscopic adhesiolysis, patients were assigned to a DPF group or a control group without DPF; the American Fertility Society (AFS) adhesion score was significantly improved and IUA presence was significantly lower in the DPF group (50).

In our study, HAG and an IUD were used after HM. Two months later, the IUD was removed and HSG was performed to evaluate the uterine cavity; IUA were observed in 4 (8%) patients.

Cell-based therapies, including platelet-rich plasma (PRP), extracellular vesicles (EVs), and stem cells, have been proposed as potential treatments for Asherman’s disease (51). PRP is a whole-blood fraction containing high platelet concentrations and multiple growth factors that may promote tissue regeneration. In a review by Wang et al. improvements were reported in recurrence of moderate-to-severe IUA (AFS score), clinical pregnancy, menstrual flow and duration, and endometrial thickness, suggesting that PRP may be a promising therapy for preventing IUA recurrence (52). Several studies have also explored stem cell therapy for endometrial regeneration in patients with Asherman syndrome (53). In the future, biomaterial-coated cell-based therapies (PRP, EVs, and stem cells) may become increasingly important for endometrial tissue regeneration (53).

Preserving fertility is a primary goal after hysteroscopic surgery. Litta et al. reported a pregnancy rate of 85.8% after HM and found no association between fibroid classification/size/number and pregnancy or delivery rates (54). Similarly, Yang et al. reported a pregnancy rate of 89.2% and a live birth rate of 70.7% (55). Ioannis et al. reported pregnancy rates of 57.1% for type 0 and 42.8% for type 1 fibroids; for type 2 fibroids, the pregnancy rate was 25% compared with 50% with expectant management (56). Bernard et al. reported a pregnancy rate of 35.5% after hysteroscopic myomectomy, which was lower than most reports (57).

In our study, pregnancy and live birth rates were 78% and 42%, respectively. While the pregnancy rate was consistent with the literature, the live birth rate was lower, which may reflect the infertile population studied and the influence of factors other than fibroids (e.g., embryo, sperm, and oocyte quality). Consistent with several reports, fibroid classification, size, and number were not associated with pregnancy or live birth rates.

Our study has several limitations. First, its retrospective design limits the strength of the conclusions and may introduce selection bias, missing data, and unmeasured confounding. Second, the sample size was relatively small (*n* = 50), which reduces statistical power and may limit generalizability. These factors should be considered when interpreting the results, and future prospective studies with larger cohorts are needed to validate and expand upon these observations. To date, no large-scale prospective study exclusively evaluating hysteroscopic myomectomy has been published. Notably, among studies focusing specifically on hysteroscopic myomectomy, two of the largest single-center retrospective cohorts were published by Al-Husban et al. (58), Catena et al. (23), each reporting outcomes in 125 patients. The available evidence base remains largely retrospective, yet, our study provides real-world data on infertile patients with FIGO type 0–II fibroids undergoing HM prior to embryo transfer within a standardized institutional pathway. Key strengths include procedural consistency and a structured postoperative strategy (physical barrier plus adhesion barrier gel, HSG-based screening, and second-look hysteroscopy when indicated). These elements may support reproducibility and inform optimization of peri-embryo-transfer management in similar IVF settings. Third, due to the institutional policy in our clinic, embryo transfer is not performed in patients with FIGO Type 0, I, or II fibroids without prior hysteroscopic myomectomy. As proceeding with embryo transfer in the presence of untreated submucosal fibroids is known to reduce implantation and pregnancy rates, assigning patients to a non-intervention control group would have been ethically inappropriate. Therefore, establishing a control group was neither ethically nor practically feasible within our clinical setting. The final limitation is that the number and the grades of embryos transferred were not evaluated in the study. These two parameters may affect IVF success, and while the pregnancy rates obtained in our study are similar to those in the literature, they may have contributed to the lower live birth rates.

Nonetheless, we evaluated pregnancy outcomes after HM while excluding major confounding factors that could independently reduce pregnancy rates.

## Conclusion

In conclusion, FIGO type 0, I, and II fibroids may reduce pregnancy outcomes in patients undergoing embryo transfer; therefore, hysteroscopic removal may be considered. Our findings support the feasibility and safety of this approach in our cohort.

## Data Availability

The data analyzed in this study is subject to the following licenses/restrictions: data can be obtained from the corresponding author. Requests to access these datasets should be directed to NP, dr.nuripeker@gmail.com.
